# Mechanical Characterization of Gypsum Composites Containing Inert and Insulation Materials from Construction and Demolition Waste and Further Application as A Gypsum Block

**DOI:** 10.3390/ma13010193

**Published:** 2020-01-02

**Authors:** Paola Villoria Sáez, Mercedes del Río Merino, Marica Sorrentino, César Porras Amores, Jaime Santa Cruz Astorqui, Carmen Viñas Arrebola

**Affiliations:** 1 Grupo de investigación TEMA, Departamento de Construcciones arquitectónicas y su control, Escuela Técnica Superior de Edificación, Universidad Politécnica de Madrid, 28040 Madrid, Spain; mercedes.delrio@upm.es (M.d.R.M.); c.porras@upm.es (C.P.A.); 2 Università degli Studi di Bari, Via Edoardo Orabona, 4, 70125 Bari BA, Italy; sor.marica@gmail.com; 3 Grupo de investigación TEMA, Departamento de Tecnología de la Edificación, Escuela Técnica Superior de Edificación, Universidad Politécnica de Madrid, 28040 Madrid, Spain; jaime.santacruz@upm.es (J.S.C.A.); carmen.vinas@upm.es (C.V.A.)

**Keywords:** plaster, recycled material, sustainable construction, interior partition, block

## Abstract

This article analyzes the feasibility of using construction and demolition waste (expanded polystyrene, ceramic, and concrete waste) in a gypsum matrix to manufacture plaster for interior coatings or for prefabricated elements for interior partitions. To do this, several gypsum specimens were prepared (4 × 4 × 16 cm) incorporating different percentages of waste based on the weight of the gypsum (25%, 50%, and 75% of ceramic, concrete, and a mixture of both). Reference samples were also produced (without additions) to compare the results obtained. The compounds with the best performance were selected and lightened by preparing other samples in which 1/3 and 2/3 of the volume of ceramic, concrete, and mixed waste were replaced with expanded polystyrene (EPS). All samples were tested in the laboratory and the following physical and mechanical characteristics were determined: density, surface hardness, flexural strength, compressive strength, capillary water absorption, and thermal conductivity. Several applications were proposed for the selected compounds. A gypsum block with a sandwich configuration was obtained (40 × 20 × 10 cm) using the optimum compound. The block was further tested regarding its density and compression strength. A comparative analysis showed that it is possible to produce materials with a gypsum matrix by adding ceramic, concrete, and EPS waste, improving the behavior of the traditional gypsum and enabling them to be applied in various construction applications. These applications have a lower environmental impact than ordinary ones because they use less primary raw material, due to the reuse of waste.

## 1. Introduction

The construction sector is one of the six components of the ecological footprint of humanity [1]. So, it is very important to rethink how to build and produce construction materials, minimizing the environmental impacts through the perspective of a circular economy. In Europe, construction and demolition waste make up one of the greatest waste flows generated, so its reuse and recycling is critical. Eurostat statistics reports that in 2016, construction and demolition waste (CDW) represented around 34% of European global waste (about 923 million tonnes) [2]. Eurostat statistics do not explain more about the composition of CDW, probably due to the lack of harmonization in the procedures used to determine the amount of CDW for each Member State (MS), and also because not all of them control waste generation flows and their treatments. However, a first approach of CDW composition across MS was published in a report by the European Commission [3], stating that the major CDW flows generated are from concrete (12–40%) and ceramic (8–54%), while the least produced involves gypsum (0.2–0.4%). In addition, the amount of waste from insulating materials is growing due to the increase of construction works aiming to improve the energy efficiency of buildings, which requires greater insulation. On the other hand, one of the most commonly used insulating materials is expanded polystyrene (EPS), which is not only used in panels for insulation but also as blocks and jack-arches in slabs to lighten construction elements.

Waste management is currently considered a priority in Europe and therefore the European Commission approved the Directive 2008/98/CE [4] which promotes waste prevention and recycling as well as the Circular Economy Action Plan which sets the basis for a circular economy. This circular model maximizes the use of all raw materials, products, and waste and achieves their maximum value, minimizing disposal and incineration practices, promoting energy savings, and thus reducing greenhouse gas emissions. In addition, Europe is also promoting CDW recovery within the Framework Program for Research and Innovation “Horizon 2020”, by promoting and funding research projects aiming to achieve zero-waste generation in Europe.

Major amounts of CDW are constantly generated and could serve as secondary raw materials if they are correctly segregated, recycled, and reused, considerably decreasing the demand for raw materials. One of the most highly recommended actions to promote zero waste and a circular economy is to decrease the waste production by applying onsite waste prevention measures (through the design of buildings) and set specific measures to guarantee waste separation on site for any unavoidable waste [5]. But what is the point of implementing onsite separation and recycling measures if there is no end use for all of this CDW? For this reason, many studies are progressively trying to replace natural raw materials with recycling materials in order to provide an end to the use of CDW.

## 2. Literature Review 

Many researchers have been concerned about the major environmental impact caused by the construction sector and have conducted many studies seeking ways to prevent and recover CDW in line with the target set by the 2009/98/CE Directive. These last years there has been an increase of research works aiming to incorporate CDW as secondary raw materials to produce recycled construction materials, such as concretes, mortars, or gypsums.

Many research works dealing with cement mortars have been conducted which incorporated different types of waste, such as ceramics and concrete [6,7,8], insulation materials [9,10,11], wood waste from demolition [12], or ladle furnace slag [13]. Most of these studies deal with recycled aggregates of concrete and ceramic waste. For example, Muñoz-Ruiperez et al. [14] analyzed lightweight masonry mortars made with expanded clay and recycled aggregates. Sáiz Martínez et al. [6] showed the technical and economic feasibility of manufacturing recycled mortars substituting 100% of the sand with recycled aggregates. Waste from insulation materials was also incorporated to reduce the density of the compounds [15]. Morales Conde et al. [12] incorporated wood waste from demolition in mortars, obtaining compounds with lower density and improved mechanical and thermal properties.

Other studies conducted in gypsum composites incorporated different waste streams (gypsum, insulation materials, plastics, wood, ceramic etc.). Pedreño-Rojas et al. [16] analyzed recycled gypsum and showed different physical proprieties in accordance with the commercial gypsum. Furthermore, San Antonio Gonzalez [17] analyzed different compounds containing polystyrene waste, resulting in lightweight gypsums with good thermal behavior and resistance to water, while the surface hardness and compression strength worsened. Moreover, Santos Jimenez et al. [18] analyzed the incorporation of ceramic waste and found that the surface hardness and water absorption by capillarity improved when less than 50% of ceramic waste was incorporated in a gypsum matrix. In general, these research works add one single type of waste and some properties improve, while others worsen. For this reason, more recent publications focus on materials incorporating a mixture of different wastes, in order to seek a synergistic effect and balance the properties obtained. Del Río Merino et al. [19] analyzed the feasibility of incorporating ceramic and extruded polystyrene waste from construction sites in two types of gypsums. Compounds with additions of 50% of ceramic waste and 1% of expanded polystyrene (EPS) can be used to produce prefabricated elements or cladding materials.

Finally, other studies were conducted to analyze not only the physical and mechanical properties of the compounds but also the final building application. For instance, Santa Cruz Astorqui et al. [20] analyzed several gypsum sandwich blocks made with two plasterboards and a lightweight core of gypsum and EPS. Several block samples were developed and tested (both solid and hollow blocks) with dimensions of 40 × 20 × 10 cm. The gypsum block was found to be lighter than the reference and kept a high degree of deformability compared with the reference, allowing a good adjustment with the deformations of the building structure. In addition, Alameda et al. [21] analyzed several gypsum plasterboards made with polyurethane foam waste and reinforced with polypropylene fibers.

While some references were found analyzing the behavior of gypsum compounds containing two types of CDW, this article analyzes the viability of incorporating three types of waste which are commonly mixed on site: concrete, ceramic, and EPS waste, and defines possible construction applications. This will result in having a smaller number of waste containers on site and simplifies CDW separation and thus its management. 

## 3. Materials and Methods

### 3.1. Materials

The materials used were:

1. Gypsum: Iberplast YG, which is produced and supplied by Placo Saint-Gobain (Madrid, Spain) is compliant with the standard EN 13279-1 [22], and is classified as type B1 by the European classification. The technical data sheet of the company reports the following characteristics:
  - Particle size: 0–2 mm
  - Surface hardness ≥ 45 Shore C units
  - Mechanical compressive resistance > 2 N/mm^2^
  - Mechanical resistance to bending > 2 N/mm^2^
  - Thermal conductivity coefficient 0.3 W/mK
2. Ceramic waste: from the gridding of bricks. The pieces of brick were struck with a hammer to obtain a size more suitable to be gridded in a crusher. The resulting product was sifted and characterized as “Coarse Aggregate” (CA) and “Fine Aggregate” (FA), which were 2 mm and 1 mm, respectively.
3. Concrete waste: obtained from a CDW recycling plant from Madrid, Spain. The resulting product was sieved and characterized as “coarse Aggregate” (CA) and “fine Aggregate” (FA), which were 2 mm and 1 mm, respectively.
4. Mixed waste: is a mixture of concrete waste (50%) and ceramic waste (50%).
5. EPS waste: were construction scarps of thermal insulation plates. The EPS plates were scratched and sieved. The final product was a particle size between 4–6 mm.

### 3.2. Experimental Plan

The experimental plan was developed using the following five phases.

#### 3.2.1. First Phase: Waste Percentages and Sample’s Elaboration

Series of three specimens (40 × 40 × 160 mm) were prepared following the regulation EN 13279-2 [23], with a water/gypsum (w/g) ratio of 0.8 and incorporating ceramics, concrete, and mixtures of both wastes in different progressive percentages: 25%wt, 50%wt, and 75%wt. In all of the cases the percentages were based on the weight of gypsum. In addition, a reference series (without waste) was also prepared in order to compare the results.

In all the series, the waste was mixed with gypsum manually and subsequently the mixture was added to the water. The samples spent 7 days at room temperature in the laboratory and were subsequently tested after drying in an oven at a constant temperature of 40 ± 2 °C for 24 h, according to standard EN 13279-2 [23]. All laboratory phases were carried out under the following conditions: a temperature of 23 ± 2 °C and an air relative humidity of around 50 ± 5%.

#### 3.2.2. Second Phase: Physical and Mechanical Tests with Compounds Containing Concrete and Ceramic Waste

**Figure 1:**
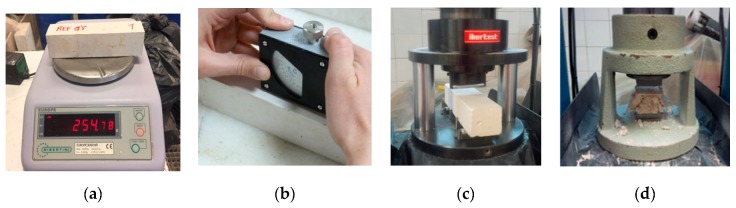
Equipment used for the tests. (**a**) Density; (**b**) Superficial hardness - Shore C; (**c**) Flexural strength; (**d**) Compression strength.

The following preliminary tests were conducted to the samples prepared in phase 1, aiming to determine their physical and mechanical characteristics: density, surface hardness Shore C, flexural, and compression strength (Figure 1). The Autotest-200/10-SW machine (Madrid, Spain) was used to test the samples for flexural and compression strengths and a Shore C durometer for the superficial hardness. For each test, the mean value achieved with the three samples was calculated and results were compared with the reference sample and with the minimum values established by the regulation, i.e., flexural and compression strength above 1 MPa and 2 MPa, respectively.

#### 3.2.3. Third Phase: Incorporation of EPS Waste and Further Tests

The best performing compounds in phase 2 were identified and selected. The ceramic, concrete, and mixed waste was substituted for by EPS waste in order to lighten the compound. The substitution of EPS was made using 1/3 and 2/3 of the aggregate in volume. Samples were made following the same procedure as that in phase 1 and were further tested regarding their density, superficial hardness, mechanical strength, thermal conductivity, and water capillarity behavior.

**Figure 2:**
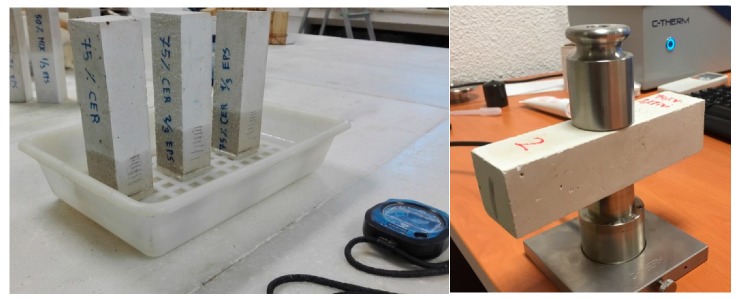
Water capillarity absorption test (**left**) and thermal conductivity test (**right**).

For the thermal behavior, the equipment Thermal Properties Analyzer from C-Therm was used to obtain the thermal conductivity coefficient values of each sample [24,25]. The measurements were taken for at least six different areas of the sample, covering all the sides except the upper face since it has a rough surface. Finally, the mean values were calculated. The water absorption by capillarity test was performed following the EN 459-2 Standard [26]. Samples were placed vertically in a container containing 1 cm of water during 10 min. Samples were weighted before and after the test and the amount of absorbed water was calculated through the difference between these measurements (Figure 2).

Also, the reduction of the raw material consumption was also examined during this stage as an index for the environmental analysis. This analysis was carried out by observing the decrease of the raw material consumption. The reduction of consumed raw material (gypsum) was analyzed for selected composites and compared with the reference sample. To this end, the quantities of each material (gypsum, wastes, and water) were used to produce the three samples, while their weight after demolding was considered. After comparing the weight of the materials used in the reference samples with those of the specimens, the reduction of the raw material employed can be calculated.

#### 3.2.4. Fourth Phase: Proposal of Building Applications

Considering the results obtained in the third phase, the optimum gypsum compounds suitable for use as gypsum-based products (coatings and gypsum elements for partition walls), were selected. For the selection, the results obtained by San Antonio González et al. [27] were used. This study conducted a survey among several experts of gypsum products in order to identify which are the key properties of gypsum to be applied as a coating or as a prefabricated element for interior partition walls. This study concluded that the surface hardness and water capillary behavior were the main characteristics valued for coatings applications, while density and compressive strength were chosen for interior partition applications. Based on these results, the following principles were considered for this study:

- The best compounds performing on surface hardness and water capillary behavior were chosen for coatings applications.
- The best compounds performing on density and compressive strength were chosen for interior partition applications (gypsum panels and blocks). A higher priority was given to the density because the material will be used to manufacture a prefabricated panel or a block and thus needs to be lightweight and easy to move and install.
- As a general criterion, compounds incorporating a higher percentage of waste and lower density were chosen, especially for prefabricated elements, in order to simplify their transport and installation.

In this way, the above considerations were used to choose the optimum compounds for each application.

#### 3.2.5. Fifth Phase: Development of A Gypsum Prefabricated Block and Tests

**Figure 3:**
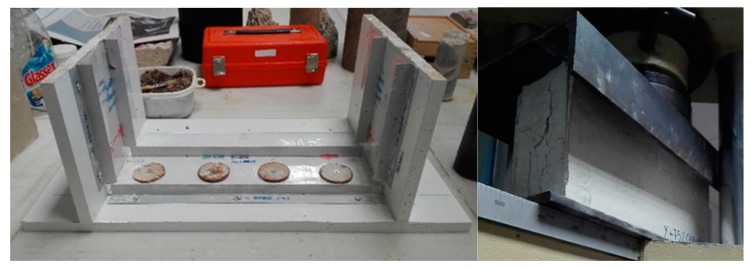
The cast used to fabricate the gypsum block (**left**) and compression test (**right**).

A gypsum block with a sandwich configuration was developed with dimensions of 40 × 20 × 10 cm. For this, gypsum laminated plasterboards were used for the external layers of the block and the core was filled with the best compound chosen in phase 4. The block was elaborated and tested following the procedure discussed in the Santa Cruz Astorqui et al. [20] study. The block was kept in a laboratory environment during 14 days before testing. The compression test was carried out on the dry block to evaluate the behavior of the partition proposed when it is exposed to an excessive deformation of the structural framework and to verify whether this type of lightweight block is viable in the construction of interior partitions (Figure 3).

While a high compressive strength is not required to an interior partition because it must only carry its dead weight, a lower stiffness is needed compared to conventional partitions. So together with the maximum compressive strength, the stiffness of the element was also analyzed. For the compression test, the universal press IBERTEST MIB-60/AM (Madrid, Spain) was used. The results obtained were compared to the ones obtained by the previous authors and with the current commercialized products.

## 4. Results and Discussion

This section shows the results obtained with the recycled gypsum compounds and suggests several applications for building construction projects.

### 4.1. Results of Phase 1: Compounds Containing Inert CDW

The results of the preliminary tests on the reference compounds and those with inert CDW (ceramics, concrete, and mix) are shown in Table 1.

Results show that the density increases with the addition of inert waste, regardless of the waste type added. It is possible to observe that the increment is around 12–13% when 25% of inert waste is added and can increase by up to 31% when adding 75% of inert waste.

For the results of superficial hardness, it is seen that in all the cases the superficial hardness increases compared to the series of reference when CDW is added. However, the percentage of increment changes with the type of waste. In fact, the surface hardness increases by about 15%, 17%, and 25% after adding ceramic waste; the increments are about 19%, 22%, and 29% for concrete waste; while the increases are about 20%, 28%, and 30% for the mix waste.

**Figure 4:**
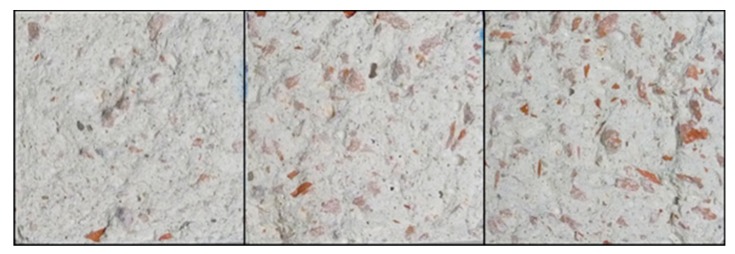
Compounds containing 25% (**left**), 50% (**center**), and 75% (**right**) of mixed waste (concrete and ceramic).

The flexural and compression strengths increase in all cases compared to the reference samples. In particular, flexural strengths increase when ceramic waste is added in percentages equal or below 50%. When higher percentages of ceramic waste are incorporated, the results slightly decrease, but are kept above the values of the reference. The initial increment in the flexural strengths may be due to the elasticity of the ceramic material, which deforms itself with the stress increment. However, higher percentages of ceramic waste result in greater surface gypsum-ceramic and thus more weakness points are created, decreasing resistance when higher percentages are added (Figure 4).

Regarding the behavior of samples containing only concrete waste, the flexural strength always decreases as the amount of waste increases, but the results always remain above the reference value. This can be explained as occurring because the concrete waste is more rigid than ceramic and thus, the gypsum compound containing concrete waste becomes much rigid as the percentage of waste increases, causing cracks to develop earlier. The mix compound presents a similar trend to the ceramic waste compounds but with lower values because of the concrete waste.

By contrast, the values for compression strength increase with the addition of concrete, ceramic, and mixed waste.

From the results obtained, the following compounds were chosen:

- Compounds containing only ceramic waste: Y0.8 + 75%CER because it has the highest amount of waste incorporated and present the best compressive strength (above 54% compared to the reference). In addition, the flexural strength is also high (above 32% compared with the reference) and diverges only 0.09 MPa from the best value achieved in compression.
- Compounds containing only concrete waste: Y0.8 + 50%CON because it presents good value for both flexural and compression strengths. The compressive strength increases by around 22% and the flexural strength increases by around 12%.
- Compounds containing a mix of concrete and ceramic waste: Y0.8 + 50%MIX of waste because it presents the best value both for the compressive and flexural strengths, with increments of 51% and 24%, respectively, compared to the reference.

These compounds were analyzed in the next phases.

### 4.2. Results of Phase 2: Selected Compounds Lightened with EPS Waste

The Table 2 shows the results obtained in this phase.

Results show that adding EPS to a gypsum compound containing ceramic and/or concrete waste results in a decrease of the density, surface hardness, and mechanical strengths. However, the values obtained with EPS are kept above the results achieved with the sample of reference (without additions). Therefore, a synergistic effect occurs when incorporating ceramic and/or concrete waste (which improve the mechanical strength and superficial hardness) and EPS waste (which reduces the density of the compounds), resulting in a compound with balanced properties and similar behavior to the gypsum of reference without recycled aggregates.

In particular, the incorporation of EPS decreases the density of the samples reaching the values of the reference samples. Also, the superficial hardness decreases with the addition of EPS, but the lowest value obtained is above the reference value (around 8% with ceramic or concrete waste and 12% with mixed waste).

The incorporation of EPS decreases the flexural strength in the samples with ceramic waste, while in that with concrete or mix waste increases with an incorporation of 1/3 of EPS and decreases with an incorporation of 2/3, possibly due to a greater elasticity of the material. The addition of EPS decreases the compressive strength of samples only with inert waste so quickly that the sample with ceramic waste and 2/3 of EPS reaches the reference value, while the sample with mix waste and 2/3 of EPS ends up near it.

The analysis of the water capillary absorption shows that the addition of EPS increases the water absorption in most specimens so that the sample with 50% of concrete waste and 2/3 of recycled EPS and the sample with 50% of mix waste and 2/3 of recycled EPS both reach the reference value in the first case and exceed it in the second. Therefore, the lightened samples have worse behavior.

For the thermal conductivity coefficient, the results show that the EPS addition decreases the values of the samples only with inert waste, improving their thermal behavior.

In general, from the results obtained in the laboratory, the compound Y0.8 + 75%CER + 1/3EPS should be highlighted as a good compound for building construction materials, because it achieves similar results to the gypsum of reference. 

Table 3 shows the results obtained in the environmental analysis to calculate the amount of raw materials consumed for each compound. The results show that the most sustainable compounds, consuming fewer raw materials, are those incorporating ceramic and EPS waste. With these compounds, the reduction of raw material consumption reaches around 10.3% in the compound with 75% being ceramic waste and 2/3 being EPS waste.

### 4.3. Results of Phase 3: Proposals for Building Applications

This section shows the analyzed compounds which are more suitable to be used as interior gypsum coatings and gypsum-based blocks for interior partition walls.

**Figure 5:**
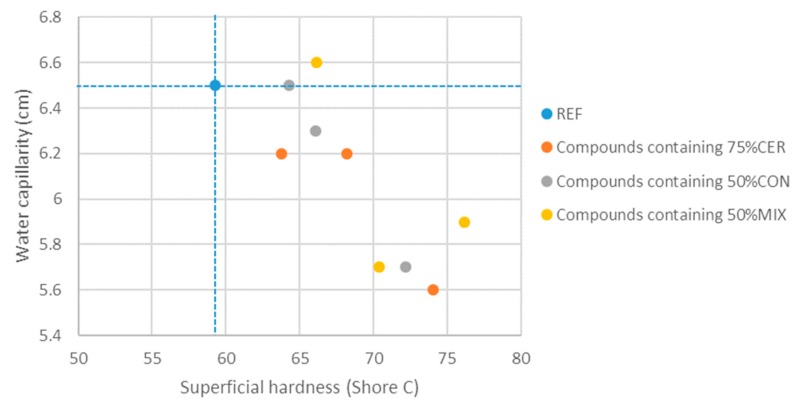
Results of superficial hardness vs. water absorption based on the capillarity of phase 2 samples.

For gypsum coatings, the values obtained in the superficial hardness and water capillarity tests were used. In this sense, Figure 5 shows the values for superficial hardness and water absorption due to the capillarity of the studied samples.

It can be seen that gypsum with 75% ceramic waste provides the best material for a coating application, presenting the second best surface hardness value and the lowest water absorption index by capillarity, with an increase by 25% of the surface hardness and a 14% reduction of water absorption. The gypsum compound can be applied directly over the untreated partition, in this case made with hollow bricks.

**Figure 6:**
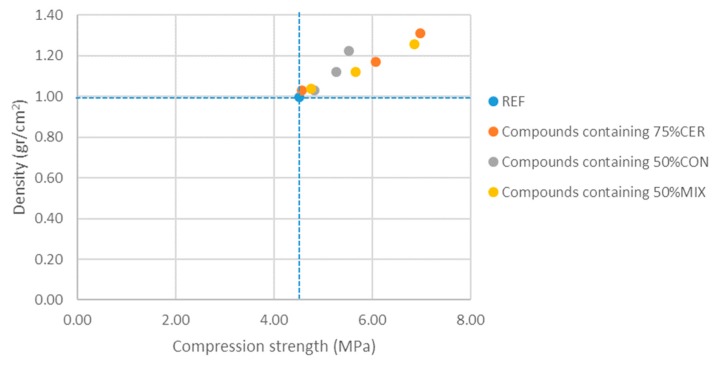
Results of density vs. compression strength of phase 2 samples.

For a gypsum-based block comprising a partition wall, the compressive strength and the density of the material are key properties. Therefore, Figure 6 shows the relationship between the density and the compressive strength of the analyzed compounds. Results show that the lighter material is the gypsum-based compound with the addition of 75% ceramic waste and with 2/3 recycled EPS.

The compound Y0.8 + 75%CER + 2/3EPS can be used to manufacture gypsum-based blocks for interior partitions.

### 4.4. Results in Phase 4: Development and Test of the Gypsum-Based Block Prototype

**Figure 7:**
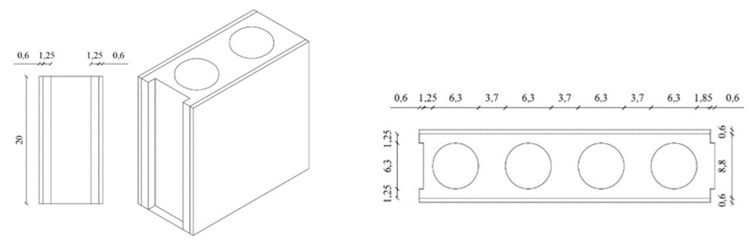
Design of the proposed hollow gypsum block with a sandwich configuration (dimensions in cm).

A hollow gypsum block with a sandwich configuration was chosen. The core of the sandwich block was filled with Y0.8 + 75%CER + 2/3EPS compound and laminated plasterboards (6 mm) were placed in the outer layers of the block, following the method of Santa Cruz Astorqui et al. [20] (Figure 7).

Table 4 shows the results obtained for the block prototype and compares the values obtained with the results obtained by previous works. Results show that the block developed is more resistant (around 50%) than a hollow block but less than the solid block or traditional partition.

**Figure 8:**
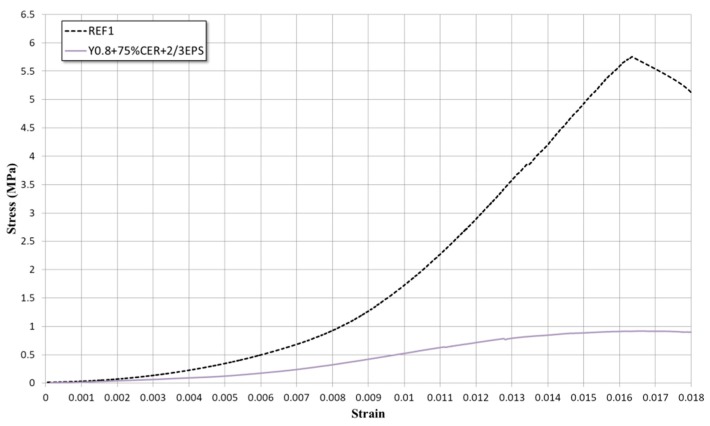
Stress-strain graph of the prototype and a traditional partition.

**Figure 9:**
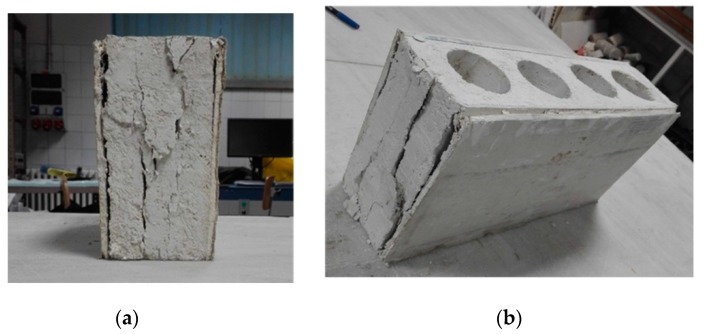
State and cracks of the block after compression test. (**a**) side of the block; (**b**) block tested

However, for an interior partition, the compression resistance is not so important because it is not a structural element. Its deformation is more relevant in order to adapt to the deformation of the building structure. The stress-strain graph shows that the block falls into the elastic zone (Figure 8). After the compression test, the fracture and breakage produced is examined. In this sense, the core of the block was separated from the plasterboards due to the insufficient adhesion between them (possibly due to the absorption of the hydration water by the cardboard). Also, cracks were observed in the core of the block, coinciding with the inner holes, which are the most fragile areas of the block. However, the rest of the core remains cohesive (Figure 9).

## 5. Conclusions

From the results obtained, it is possible to conclude that it is viable to incorporate mixes of concrete, ceramic, and EPS waste in gypsum composites for building interior coatings or prefabricated elements. In particular, the following conclusions were reached:

- When considering mechanical properties, the addition of ceramic and concrete waste improves mechanical behavior. In particular, the incorporation of ceramic waste (until reaching 75% over the gypsum weight) increases the mechanical strengths and the density of the gypsum without additions.
- Adding EPS waste in compounds containing ceramic and concrete waste decreases their density, improving the thermal behavior and decreasing the water capillarity absorption.
- Incorporating ceramic, concrete, and EPS waste in gypsum creates a synergy between the materials, because the composites obtained achieve similar or improved properties compared to traditional gypsums without additions.
- Gypsums containing inert and EPS waste reduce the environmental impact of the traditional gypsums due to the reduction of raw materials consumption (around 7.5% when inert waste is incorporated and up to 10.5% when EPS is also added).
- The composite Y0.8 + 75%CER is suitable for coating application because it presents the best surface hardness value with the lowest water absorption by capillarity. Moreover, the composite Y0.8 + 75%CER + 2/3EPS is appropriate to produce hollow blocks due to its density and compression strength, which is similar to the gypsum of reference.
- A sandwich gypsum block filled with Y0.8 + 75%CER + 2/3EPS compound and laminated plasterboards (6 mm) as external elements was found to be more resistant than currently commercialized hollow blocks.

In general, these compounds provide direct advantages, as the mechanical and thermal behavior is improved compared to traditional gypsums, while the consumption of raw materials is reduced. Additionally, further work should be made focusing on other building applications as well as other forms of environmental analysis, such as life cycle analysis.

## Figures and Tables

**Table 1:** Results obtained in phase 1.

Series	Compound	Density (gr/cm^3^)	Superficial Hardness (Shore C)	Flexural Strength (MPa)	Compressive Strength (MPa)
1	YG0.8 REF	1.00	59.26	2.61	4.52
2	YG0.8 + 25%CER	1.12	67.96	2.96	5.01
3	YG0.8 + 50%CER	1.22	69.03	3.45	6.27
4	YG0.8 + 75%CER	1.31	74.03	3.36	6.98
5	YG0.8 + 25%CON	1.13	70.30	3.06	5.47
6	YG0.8 + 50%CON	1.23	72.16	2.91	5.53
7	YG0.8 + 75%CON	1.31	76.70	2.84	6.21
8	YG0.8 + 25%MIX	1.12	71.23	2.95	5.46
9	YG0.8 + 50%MIX	1.26	76.13	3.24	6.86
10	YG0.8 + 75%MIX	1.31	77.86	3.12	6.75

**Table 2:** Results obtained in phase 2.

Series	Compound	Density (gr/cm^3^)	Superficial Hardness (Shore C)	Flexural Strength (MPa)	Compression Strength (MPa)	Water Capillarity (cm)	Thermal Conductivity (W/mK)
1	YG0.8 REF	1.00	59.26	2.61	4.52	6.50	0.27
4	YG0.8 + 75%CER	1.31	74.03	3.36	6.98	5.60	0.40
4.A	YG0.8 + 75%CER-1/3 EPS	1.17	68.20	3.04	6.07	6.20	0.29
4.B	YG0.8 + 75%CER-2/3 EPS	1.03	63.80	2.90	4.56	6.20	0.28
6	YG0.8 + 50%CON	1.23	72.16	2.91	5.53	5.70	0.36
6.A	YG0.8 + 50%CON-1/3 EPS	1.12	66.10	2.96	5.26	6.30	0.33
6.B	YG0.8 + 50%CON-2/3 EPS	1.03	64.26	2.84	4.83	6.50	0.33
9	YG0.8 + 50%MIX	1.26	76.13	3.24	6.86	5.90	0.40
9.A	YG0.8 + 50%MIX-1/3 EPS	1.12	70.36	3.36	5.67	5.70	0.35
9.B	YG0.8 + 50%MIX-2/3 EPS	1.04	66.13	3.05	4.76	6.60	0.35

**Table 3:** Quantities of materials used to produce the samples and the reduction of raw materials consumption compared to the reference sample.

Compound	Materials	Compound for One Cast		One Sample (4 × 4 × 16 cm^3^)	Reduction of Raw Material (%)	
		Weight (g)	%	Weight (g)		
Y 0.8	Gypsum	1000	55.56%	142.02	–	
	Water	800	44.44%	113.61	–	
	Ceramic waste	0	0.00%	0.00	–	–
	Concrete waste	0	0.00%	0.00	–	–
	EPS waste	0	0.00%	0.00	–	–
Y 0.8 + 75%CER	Gypsum	1000	39.22%	131.41	7.5%	
	Water	800	31.37%	105.13	7.5%	
	Ceramic waste	750	29.41%	98.56	–	–
	Concrete waste	0	0.00%	0.00	–	–
	EPS waste	0	0.00%	0.00	–	–
Y 0.8 + 75%CER + 1/3EPS	Gypsum	1000	43.38%	130.17	8.3%	
	Water	800	34.70%	104.13	8.3%	
	Ceramic waste	499.9	21.68%	65.07	–	–
	Concrete waste	0	0.00%	0.00	–	–
	EPS waste	5.4	0.23%	0.70	–	–
Y 0.8 + 75%CER + 2/3EPS	Gypsum	1000	48.53%	127.40	10.3%	
	Water	800	38.82%	101.92	10.3%	
	Ceramic waste	249.8	12.12%	31.83	–	–
	Concrete waste	0	0.00%	0.00	–	–
	EPS waste	10.8	0.52%	1.38	–	–
Y 0.8 + 50%CON	Gypsum	1000	43.48%	136.43	3.9%	
	Water	800	34.78%	109.15	3.9%	
	Ceramic waste	0	0.00%	0.00	–	–
	Concrete waste	500	21.74%	68.22	–	–
	EPS waste	0	0.00%	0.00	–	–
Y 0.8 + 50%CON + 1/3EPS	Gypsum	1000	46.62%	133.08	6.3%	
	Water	800	37.29%	106.46	6.3%	
	Ceramic waste	0	0.00%	0.00	–	–
	Concrete waste	342.4	15.96%	45.57	–	–
	EPS waste	2.7	0.13%	0.36	–	–
Y 0.8 + 50%CON + 2/3EPS	Gypsum	1000	50.76%	134.21	5.5%	
	Water	800	40.61%	107.37	5.5%	
	Ceramic waste	0	0.00%	0.00	–	–
	Concrete waste	164.4	8.35%	22.06	–	–
	EPS waste	5.6	0.28%	0.75	–	–
Y 0.8 + 50%MIX	Gypsum	1000	43.48%	139.88	1.5%	
	Water	800	34.78%	111.91	1.5%	
	Ceramic waste	250	10.87%	34.97	–	–
	Concrete waste	250	10.87%	34.97	–	–
	EPS waste	0	0.00%	0.00	–	–
Y 0.8 + 50%MIX + 1/3EPS	Gypsum	1000	46.86%	134.60	5.2%	
	Water	800	37.49%	107.68	5.2%	
	Ceramic waste	166.5	7.80%	22.41	–	–
	Concrete waste	164.4	7.70%	22.13	–	–
	EPS waste	3.1	0.15%	0.42	–	–
Y 0.8 + 50%MIX + 2/3EPS	Gypsum	1000	50.72%	134.43	5.3%	
	Water	800	40.57%	107.54	5.3%	
	Ceramic waste	83.3	4.22%	11.20	–	–
	Concrete waste	82.2	4.17%	11.05	–	–
	EPS waste	6.3	0.32%	0.85	–	–

**Table 4:** Comparison of compression strengths and weights of the block developed, traditional partition and two gypsum blocks currently used in the market [20].

	Compression Strength (MPa)	Weight Per m^2^ (kg)
Prototype developed. Hollow gypsum block with a sandwich configuration	0.95	67.06
Gypsum solid block	> 5.00	96.00
Gypsum hollow block	0.63	75.00
Traditional partition (ceramic brick and plaster)	2.89	81.60

## References

[1] McLellan R., Iyengar L., Jeffries B., Oerlemans N. (2014). Living Planet. Report 2014: Species and Spaces, People and Places.

[2] Eurostat Eurostat Statistics for Waste Flow Generation 2016. http://epp.eurostat.ec.europa.eu/portal/page/portal/eurostat/home/.

[3] Monier V., Hestin M., Trarieux M., Mimid S., Domröse L., Van Acoleyen M., Hjerp P., Mudgal S. (2011). Study on the Management of Construction and Demolition Waste in the EU.

[4] Commision E. (2008). Directive 2008/98/EC of the European Parliament and of the Council on Waste. Off. J. Eur. Union.

[5] Osmani M., Villoria Sáez P. (2019). Current and emerging construction waste management status, trends and approaches. Waste.

[6] Saiz Martínez P., González Cortina M., Fernández Martínez F., Rodríguez Sánchez A. (2016). Comparative study of three types of fine recycled aggregates from construction and demolition waste (CDW), and their use in masonry mortar fabrication. J. Clean. Prod..

[7] Jiménez J., Ayuso J., López M., Fernández J., De Brito J. (2013). Use of fine recycled aggregates from ceramic waste in masonry mortar manufacturing. Constr. Build. Mater..

[8] Lee S.-T., Swamy R.N., Kim S.-S., Park Y.-G. (2008). Durability of mortars made with recycled fine aggregates exposed to sulfate solutions. J. Mater. Civ. Eng..

[9] Zia K.M., Bhatti H.N., Bhatti I.A. (2007). Methods for polyurethane and polyurethane composites, recycling and recovery: A review. React. Funct. Polym..

[10] Arroyo Sanz R. (2017). Addition of New Polymerbased and Mineral-Based Fillers in Mortar: Influences of Polyurethane and Afwillite on the Microstructure and Final Properties.

[11] Piña Ramírez C., del Río Merino M., Viñas Arrebola C., Vidales Barriguete A., Kosior-Kazberuk M. (2019). Analysis of the mechanical behaviour of the cement mortars with additives of mineral wool fibres from recycling of CDW. Constr. Build. Mater..

[12] Morales-Conde M., Rubio-de-Hita P., Pérez-Gálvez F. (2017). Composite Mortars Produced with Wood Waste from Demolition: Assessment of New Compounds with Enhanced Thermal Properties. J. Mater. Civ. Eng..

[13] Rodríguez Sáiz Á., Manso J.M., Aragón Á., González J.J. (2009). Strength and workability of masonry mortars manufactured with ladle furnace slag. Resour. Conserv. Recycl..

[14] Muñoz-Ruiperez C., Rodríguez A., Gutiérrez-González S., Calderón V. (2016). Lightweight masonry mortars made with expanded clay and recycled aggregates. Constr. Build. Mater..

[15] Arroyo R., Horgnies M., Junco C., Rodríguez A., Calderón V. (2019). Lightweight structural eco-mortars made with polyurethane wastes and non-Ionic surfactants. Constr. Build. Mater..

[16] Pedreño-Rojas M., Flores-Colen I., De Brito J., Rodríguez-Liñán C. (2019). Influence of the heating process on the use of gypsum wastes in plasters: Mechanical, thermal and environmental analysis. J. Clean. Prod..

[17] San-Antonio-González A., Merino M.D.R., Arrebola C.V., Villoria-Sáez P. (2015). Lightweight material made with gypsum and EPS waste with enhanced mechanical strength. J. Mater. Civ. Eng..

[18] Santos Jiménez R., San-Antonio-González A., Del Río Merino M., González Cortina M., Viñas Arrebola C. (2015). Gypsum Composites with CDW as Raw Material. World Acad. Sci. Eng. Technol. Int. J. Civ. Environ. Struct. Constr. Archit. Eng..

[19] Del Río Merino M., Santa Cruz Astorqui J., Villoria Sáez P., Santos Jiménez R., González Cortina M. (2018). Eco plaster mortars with addition of waste for high hardness coatings. Constr. Build. Mater..

[20] Santa-Cruz-Astorqui J., Del-Río-Merino M., Villoria-Sáez P., Porras-Amores C. (2019). Analysis of the viability of prefabricated elements for partitions manufactured with plaster and EPS from waste recycling. DYNA Ingeniería Ind..

[21] Alameda L., Calderón V., Junco C., Rodríguez A., Gadea J., Gutiérrez-González S. (2016). Characterization of gypsum plasterboard with polyurethane foam waste reinforced with polypropylene fibers. Mater. Constr..

[22] UNE (2009). Gypsum Binders and Gypsum Plasters. Part 1: Definitions and Requirements.

[23] NSAI (2006). Gypsum Binders and Gypsum Plasters. Part 2: Test Methods.

[24] Cha J., Seo J., Kim S. (2012). Building materials thermal conductivity measurement and correlation with heat flow meter, laser flash analysis and TCi. J. Therm. Anal. Calorim..

[25] Porras Amores C., Santa Cruz Astorqui J., Del Rio Merino M., Villoria Sáez P., Viñas Arrebola C. (2019). Thermal behavior of traditional lightweight gypsum with construction and demolition waste materials. DYNA.

[26] UNE (2011). Building Lime—Part 2: Test Methods.

[27] San Antonio González A.D. (2017). Caracterización de Compuestos Eco-Eficientes de Yeso Aligerado con Residuo de Poliestireno Extruido (XPS). Ph.D. Thesis.

